# The effect of boldness on decision-making in barnacle geese is group-size-dependent

**DOI:** 10.1098/rspb.2010.2266

**Published:** 2010-12-01

**Authors:** Ralf H. J. M. Kurvers, Vena M. A. P. Adamczyk, Sipke E. van Wieren, Herbert H. T. Prins

**Affiliations:** Resource Ecology Group, Wageningen University, Droevendaalsesteeg 3a, 6708 Wageningen, The Netherlands

**Keywords:** boldness, decisions, group size, information, personality, quorum sensing

## Abstract

In group-living species, decisions made by individuals may result in collective behaviours. A central question in understanding collective behaviours is how individual variation in phenotype affects collective behaviours. However, how the personality of individuals affects collective decisions in groups remains poorly understood. Here, we investigated the role of boldness on the decision-making process in different-sized groups of barnacle geese. Naive barnacle geese, differing in boldness score, were introduced in a labyrinth in groups with either one or three informed demonstrators. The demonstrators possessed information about the route through the labyrinth. In pairs, the probability of choosing a route prior to the informed demonstrator increased with increasing boldness score: bolder individuals decided more often for themselves where to go compared with shyer individuals, whereas shyer individuals waited more often for the demonstrators to decide and followed this information. In groups of four individuals, however, there was no effect of boldness on decision-making, suggesting that individual differences were less important with increasing group size. Our experimental results show that personality is important in collective decisions in pairs of barnacle geese, and suggest that bolder individuals have a greater influence over the outcome of decisions in groups.

## 1. Introduction

The benefits of group living for individual group members are well established and include reduced predation risk and increased sharing of information [1]. If the benefits of grouping outweigh the costs of splitting, animals with conflicting interest may face situations where they have to reach consensus decisions, whereby they have to choose collectively between various alternatives (see [2] for a review); for instance, choosing between different movement directions. Coordinated behaviour in such groups might arrive as a result of communal decisions (‘democracy’) [3] or by following decisions of other individuals—so-called ‘leaders’ (‘despotism’) [4,5]. Leadership has been explained by individual variation in dominance ([6–8]; but see [9]), motivation [10–12], relatedness [8,13] and social relations [8,14]. Information might also be an important component for determining leadership. Providing a few individuals in a large crowd of humans with information can result in collective movements led by the informed humans [15]. Likewise, a minority of informed golden shiners, *Notemigonus crysoleucas* [16], were able to lead groups towards a food source. Also, honeybee [17] and ant [18] migrations are led by a minority of informed individuals. When observing social information, individuals need to weigh this information against their personal information and an important mechanism in mediating this balance is quorum sensing. Quorum sensing implies that the probability of an individual performing a certain behaviour increases as a function of the number of conspecifics already demonstrating this behaviour [19]. Individuals only follow information if they observe a certain threshold (or majority) of individuals performing a particular behaviour. Empirical examples include ants [20], African buffalo (*Syncerus caffer* [3]) and three-spined sticklebacks (*Gasterosteus aculeatus*), where one replica conspecific was able to control the movement of a solitary individual but not of larger groups [21].

Recently, the role of personality in contributing to leadership has been acknowledged, and this might therefore also directly influence collective movements. Personality describes the phenomenon of differences among individuals of the same species in behavioural and physiological traits being consistent over time and context [22–25]. Examples in guppies (*Poecilia reticulata* [26]), three-spined sticklebacks [27], barnacle geese (*Branta leucopsis* [28]) and zebra finches (*Taenopygia guttata* [29,30]) show that bolder individuals are more often found in the leading edge of moving groups. Although the role of personality in determining leadership is quite well established, the relationship between personality and collective decisions in groups remains poorly understood.

Here, we studied whether the personality of an individual affects the way it reacts to different numbers of informed individuals and how this in turn affects collective group movements in barnacle geese. Barnacle geese are highly gregarious birds forming large flocks during foraging, roosting and migration. Boldness has been shown to be a good proxy for personality in barnacle geese [28,31,32]. We introduced naive barnacle geese together with either one or three informed individuals (all of intermediate boldness level) in a labyrinth and studied the decision-making process in these groups (i.e. whether naive individuals decided where to go for themselves). Based on the observation that bolder barnacle geese walk in front towards a food source more often in pairs of geese as compared with shyer individuals [28], and that bold/fast individuals are less reactive to companions than shy/slow individuals (great tits, *Parus major* [33]; ravens, *Corvus corax* [34]; and three-spined sticklebacks, [27]), we expected that bolder individuals would make a decision on their own more often, whereas shyer individuals were expected to wait more often for the decision of the informed individual(s) and follow this decision.

## 2. Material and methods

### (a) Experimental subjects

We used captive-born wing-clipped barnacle geese (*n* = 42), each fitted with a uniquely coded leg ring for identification. Birds were sexed by cloacal inspection (20 females, 22 males). We measured tarsus and culmen length (to the nearest 0.1 mm) using callipers and wing length (1.0 mm) using a ruler. One observer carried out all measurements to minimize observer biases. Prior to decision experiment 1, we measured body mass on a digital balance (1.0 g). We used a principal component (PC) analysis of tarsus, culmen and wing lengths to derive a measure of structural body size. PC1 explained 75.6 per cent of the variation. Body condition was calculated as the residuals from a linear regression of body mass on PC1 (*R*^2^ = 0.22, *F*_1,41_ = 11.4, *p* = 0.002). When not used for the experiment, all geese were kept as one group in an outdoor aviary of 12 × 15 m at The Netherlands Institute of Ecology in Heteren, The Netherlands. Throughout the experiments, geese were fed *ad libitum* with a mixture of grains and pellets. A pond (6 × 1 m) was present in the aviary, with continuous flowing water for bathing and drinking.

### (b) Boldness

**Figure 1.**
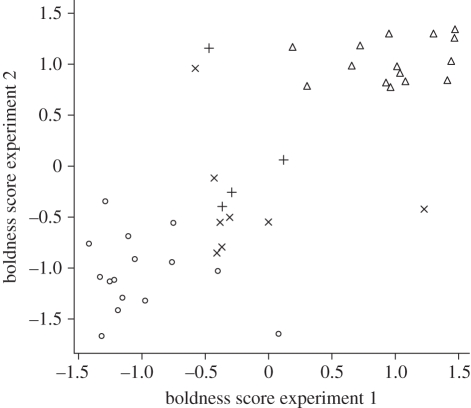
Relationship between the outcome of two novel object tests for all individuals (*n* = 42). Circles represent geese that were classified as shy (*n* = 15). Triangles represent geese that were classified as bold (*n* = 15). Pluses represent geese that were used as demonstrators in experiments 1 and 2 (*n* = 4). Crosses represent geese that were used as demonstrators in experiment 2 only (*n* = 8).

We used a novel object test to assess the boldness of all individuals (see for details [28]). We habituated individuals to an experimental arena. After habituation, we placed a novel object in the middle of the arena, introduced each goose for 10 min and scored the minimal distance (centimetre) reached between the goose and the novel object, as well as the approach latency (defined as the time elapsed (second) before the goose came within 50 cm of the novel object). Each individual was tested twice in November or December 2008 (see [28,31]). We calculated PCs of the test variables for each test as an independent measure of novel object score. PC1 explained 86.9 and 89.6 per cent of the variation for test 1 and test 2, respectively. The correlations of both the minimal distance and the approach latency with PC1 were negative, implying that high values of PC1 correspond to bolder individuals. Repeatability of novel object score was high (0.82), indicating that individuals differed consistently in their boldness scores (see also figure 1).

For the first decision experiment (see below), we selected the 15 boldest and the 15 shyest individuals as naive, focal individuals (figure 1), because we expected the largest differences in reaction towards an informed demonstrator between bold and shy individuals. We selected five intermediate individuals as demonstrators (see below). All remaining individuals (*n* = 7) were placed in a separate aviary. For the second decision experiment, we took 12 intermediate individuals as demonstrators.

### (c) Decision experiment

**Figure 2.**
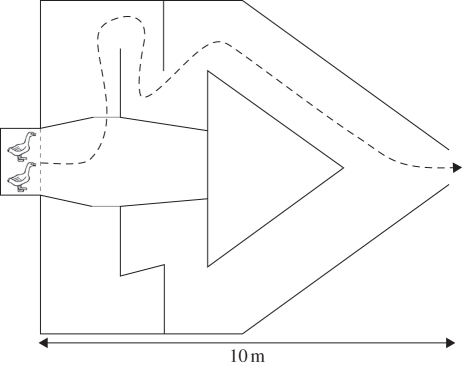
Schematic overview of the labyrinth used for the decision experiment. The black closed lines represent a wire, fenced with black anti-root cloth. The bottom of the arena was also covered with anti-root cloth. The dashed grey line represents a Plexiglas partition that was lifted 2 min after introduction of the geese. The dashed arrow represents the route that individuals had to take to arrive at the exit of the arena. The grey lines represent fictive lines. Crossing either line was used as the criterion for choosing a side.

To study the effect of personality on the decision-making process, we used a labyrinth consisting of a starting area with two identical, mirrored corridors (figure 2). One corridor led to the end of the labyrinth and back to the home aviary, whereas the other corridor led towards a dead end. Individuals were walked gently towards a wooden pen that served as the entrance of the labyrinth (figure 2). Individuals were held for 2 min in the pen before introducing them to the arena by lifting a Plexiglas partition (from outside the experimental area to minimize disturbance).

For experiment 1, we trained five individuals (of intermediate boldness levels; see also figure 1) in the route through the labyrinth. First, we introduced all five individuals (hereafter called demonstrators) together, then in groups of two individuals and finally alone. The training period lasted 5 days with three training sessions (introductions) per demonstrator per day. One demonstrator did not learn the route, leaving a total of four demonstrators.

The experimental period lasted 10 days (1–10 May 2009). Each day we introduced each demonstrator three times on its own. If they chose the correct corridor three times, and if they had also correctly chosen three times the previous day, we performed one experimental run together with a naive individual. The average success rate of the demonstrators over the 10 days was 95 per cent. For the first experimental run of each demonstrator, we randomly picked a naive individual from either the bold or the shy group. After that we alternated between a shy and a bold companion. In total, we ran 29 trials. One shy companion was not used in the experiment since it showed unusual behaviour in the home aviary (such as fleeing from the group and trying to jump over fences). One trial was excluded from the analysis since the naive individual (from the bold group) managed to escape from the labyrinth, leaving a total of 28 successful trials, 14 with a bold individual and 14 with a shy individual. Demonstrators were used between six and eight times each.

During each trial, we scored (i) whether the naive individual was the first to enter either of the corridors (yes/no; see also figure 2) and, (ii) if the naive individual was not the first to enter a corridor, whether the individual followed the demonstrator (yes/no). Following was defined as entering the same corridor within 5 s after the demonstrator.

Experiment 2 was performed eight months later. In this experiment, we used groups of four individuals, containing three informed demonstrators and one naive individual. During the training phase, we trained four groups of three demonstrators. Since four of these 12 individuals were already used in experiment 1 as demonstrators and were possibly more experienced, we assigned one of these four individuals to each demonstrator group to minimize variation between demonstrator groups. We trained the four demonstrator groups three times a day for a period of 4 days, after which the experimental period started. The experimental period lasted 9 days (20–28 January 2010) and we used demonstrator groups between five and eight times following the same criteria and the same experimental protocol as in experiment 1. We used the same 28 naive individuals as in experiment 1, except for one shy individual that had died in the period between experiments. This individual was replaced by another shy individual. We slightly rebuilt the arena (but keeping the same dimensions) to avoid recognition of the arena by the naive individuals. During each trial, we scored (i) whether the naive individual was the first to enter either of the corridors (yes/no), and, if the naive individual was not the first to enter a corridor, (ii) whether it followed the demonstrator(s) (yes/no) and (iii) which position in the group it occupied when entering a corridor.

### (d) Dominance

Since dominance might affect collective movements, we established the dominance hierarchy for experiment 1 by scoring agonistic interactions within dyads in the flock of 34 individuals (15–28 May 2009). In total, we scored 1185 interactions (mean number per individual: 69.7; range: 28–193 interactions). The value of Kendall's linearity index (*k* = 0.48), Landau's index and the corrected index of the sociometric matrix (*h* = 0.48, *h*′ = 0.51) were moderate. We constructed a linear dominance hierarchy, but to evaluate whether this hierarchy reflected the pair-wise dominance relationships experienced between individuals in a pair, we also tested the dominance in all combinations of pairs used during decision experiment 1. We introduced each pair in an arena (1 × 3 m), offering a small patch of grass (30 × 20 cm). For 30 min, we scored each agonistic interaction (22–26 May 2009). The average number of interactions was 9.7 per trial (range: 0–35), and all agonistic interactions except two were unidirectional. In 23 out of 28 trials, we observed agonistic interactions. Of the winners, 22 out of 23 corresponded to the linear dominance hierarchy, indicating that the position in the linear dominance hierarchy is a good predictor for the pair-wise dominance. For these 23 pairs, we used the outcome during the pair-wise interaction to establish the dominance; for the remaining five pairs we used the linear dominance hierarchy to establish which individual of the pair was dominant.

### (e) Statistics

To test whether boldness affected an individual's decision to choose a corridor prior to the demonstrator we used ‘naive individual first to choose a corridor’ (yes/no) as a response variable in a generalized linear mixed model with binomial errors and a logit link function. As fixed effects, we fitted boldness score of the naive individual (on a continuous scale), dominance (dominant/subordinate), body condition (continuous) and sex (male/female). For experiment 1, we also included the boldness score of the demonstrator as a fixed effect to control for a possible effect of boldness differences between demonstrators. We constructed separate models for experiments 1 and 2. Prior to the mixed model analysis, we used Spearman's rank correlations to study possible correlations between body condition, dominance rank and boldness. To test whether there were sex differences in boldness score, body condition or dominance we used non-parametric Mann–Whitney *U-*tests. There were no significant correlations between dominance rank, body condition and boldness (all |*r*_*s*_| < 0.17, all *p* > 0.3, *n* = 34). There were no significant differences between males and females in boldness or body condition (all *U* > 110, *U*_1_ = 18, *U*_2_ = 16, all *p* > 0.4). Males, however, had a higher dominance rank than females (*U* = 39, *p* < 0.001). Since sex and dominance were not independent, we constructed three separate models, including (i) all terms, (ii) all terms but excluding sex, and (iii) all terms but excluding dominance, to study the effect of sex and dominance separately.

Since it has been shown that personality traits might affect the behaviour of other individuals (e.g. [27,28]), we analysed whether the boldness score of the naive individual affected the decision time of the demonstrator in experiment 1, using all trials in which the demonstrator entered a corridor first. Likewise, we analysed whether the boldness score of the demonstrator affected the decision time of the naive individual, using all trials in which the naive individual entered a corridor first.

To minimize pseudoreplication, we included demonstrator identity as a random effect in all mixed models. We started with full models containing all terms. Minimal adequate models were obtained by stepwise deletion of non-significant terms (*p* > 0.1), starting with the least significant term. To compare the explanatory power of two subsequent models, we used a log-likelihood ratio test that follows a *χ*^2^ distribution, with degrees of freedom equal to the difference in the number of parameters between the two models. We present these *χ*^2^-values and *p*-values as well as the estimate (est.) and standard errors (s.e.) of the individual factors. In addition, we performed a separate analysis entering all fixed effects independent of one another. We used the package lme4 for mixed model procedures in R (v. 2.11.1).

## 3. Results

**Figure 3.**
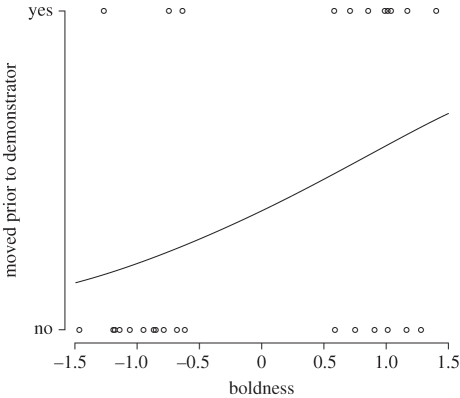
The probability of a naive individual choosing a side prior to the informed demonstrator increased with increasing boldness score of the naive individual during experiment 1 in groups with one informed demonstrator. The line is a logit regression line.

For trials with one informed individual, the probability of a naive individual to choose a side prior to the demonstrator increased with increasing boldness score (est. = 0.87, s.e. = 0.45, 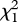 = 3.97, *p* = 0.046; figure 3). There was no significant effect of dominance, body condition, sex or boldness score of the demonstrator (dominance: est. = 1.65, s.e. = 1.20, 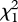 = 2.22, *p* = 0.14; body condition: est. = 0.002, s.e. = 0.002, 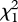 = 1.68, *p* = 0.20; sex: est. = 0.42, s.e. = 0.81, 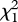 = 0.25, *p* = 0.62; boldness of demonstrator: est. = 0.38, s.e. = 1.78, 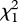 = 0.04, *p* = 0.84). All three models (i.e. full model, excluding sex and excluding dominance) gave similar results, indicating that neither dominance nor sex was significant. Likewise, we arrived at similar results when we entered factors independent of each other. When a demonstrator entered a corridor first, it always chose the correct route and in nearly all cases the naive individual followed the demonstrator (*n* = 15/17). When a naive individual moved first there was no preference for either corridor (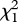 = 2.3, *p* = 0.13, *n* = 11), indicating that naive individuals did not have side preferences. In these trials, the informed demonstrator always followed if the naive individual entered the correct corridor. If the naive individual entered the incorrect corridor, in two out of three cases the informed demonstrator waited for the naive individual to return to the entrance of the arena before walking together to the correct side, and in only one case the dyad split and chose different routes, indicating the strength of group cohesion in this species. The decision time of the demonstrator (mean ± s.e. = 16.5 ± 5.9 s, *n* = 17) was not affected by the boldness score of the naive individual (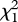 = 0.23, *p* = 0.62). Likewise, the decision time of the naive individual (mean ± s.e. = 8.7 ± 2.3 s, *n* = 11) was not affected by the boldness score of the demonstrator (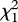 = 0.23, *p* = 0.63).

For trials with three informed individuals, an informed demonstrator chose to move through the maze first on all but two occasions (*n* = 26/28), and was always seen to choose the correct route. There was no effect of boldness score on the probability of moving first (est. = 0.014, s.e. = 0.74, 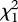 = 0, *p* = 0.98), nor was there an effect of body condition or sex (body condition: est. = 0.0004, s.e. = 0.003, 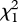 = 0.02, *p* = 0.89; sex: est. = 0, s.e. = 0, 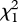 = 0, *p* = 1). Entering factors independent of each other gave similar results. When a demonstrator moved first, the naive individual always followed the demonstrator. In most trials (*n* = 22/28), the naive individual was walking either at the third or fourth position in the group and there was no effect of the boldness score of the naive individual on the position it occupied in the group (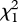 = 0.09, *p* = 0.76).

## 4. Discussion

As expected, the probability of moving first increased with increasing boldness score, although this effect was dependent on the number of geese: in pairs of geese boldness affected decision-making, whereas in groups of four individuals there was no effect of boldness. There is little known about the extent to which personality affects collective decision-making. In foraging groups of sheep, bold and shy individuals show different spatial distribution patterns over resource patches, with shy individuals showing a lower tendency to split into smaller subgroups than bold individuals [35]. The observed patterns of spatial distribution have been explained by individual variation in social attraction that results in emerging collective choices through the nonlinear dynamics of interactions between individuals of different boldness levels [36]. Likewise, in fish, shy individuals have a higher shoaling tendency [26,37,38]. As well as a higher sociability, shy individuals also have a lower tendency to explore by themselves, which is confirmed by several studies showing that mainly bolder individuals take the role of leader [26–30]. Here, we show that in pairs the probability of waiting for the decision of an informed conspecific decreased with increasing boldness score of the naive individual, suggesting that bolder individuals have a greater influence over the outcome of decisions in groups. In barnacle geese, boldness is not correlated with either activity or exploration of a novel environment [28]; hence, our results cannot be explained by differences in activity levels between individuals, nor by differences in exploration rate of a novel environment.

To what extent personality traits measured in isolation have consequences for behavioural differences between individuals in groups is important for understanding the impact of personality differences in an ecological context. In groups, individual behaviour can be affected both by individual differences like personality [31,39], but also by social influences [26,28,33,39]. In larger groups, the feedback and interaction processes between traits may alter and it has been hypothesized that individual behavioural differences (owing to, for example, personality traits) in larger groups become more pronounced (by self-organization [40]) or, in contrast, become less pronounced (by consensus decisions [41]). Our results show that personality differences were important in pairs of geese, but not in groups of four individuals, suggesting that individual differences became less important with increasing group size. However, the two experiments differed in an important aspect, which forces us to be cautious about a direct comparison between the experiments. In experiment 1, there was only one informed individual, whereas in experiment 2 there were three informed individuals. A key difference is the number of informed individuals, and this could have consequences if geese use a form of quorum sensing. The number of individuals demonstrating the correct entrance was higher in experiment 2 than in experiment 1, and this might help explain our observation that boldness was important in pairs (with one informed individual) but not in groups of four individuals (with three informed individuals). Most naive individuals in experiment 2 were walking either in third or fourth position, which seems to suggest that the probability of following increased with the number of individuals entering a certain corridor. Whether this is due to a minimum threshold or a majority rule (see also [42]) cannot be discriminated in our experiments. Careful experimental manipulation of group size, number of informed individuals and boldness differences could allow one to investigate this matter further and explore whether individuals differing in boldness follow similar quorum-sensing rules, or whether boldness differences also result in different quorum-sensing rules. Nonetheless, the observation that individual variation in boldness did not result in behavioural differences in groups with three informed individuals (whereas it did in groups with one informed group-mate) highlights the need to study the expression of personality in larger, more natural groups. For instance, most studies showing that personality affects leadership are done in very small groups (e.g. pairs [27–30]). An evaluation of the expression of personality in larger, more natural groups is important to understand the significance of personality in a natural situation.

Nomakuchi *et al*. [43] performed a similar experiment to ours in which they trained three-spined sticklebacks to follow a route through a maze and introduced these informed individuals together with naive individuals differing in exploration score. They found that more explorative individuals followed the informed individual to a larger extent than less explorative individuals. Unfortunately, they used the same maze to study exploration behaviour and following behaviour, making it difficult to assess whether the increased tendency to follow demonstrators by more explorative individuals is not a result of an increased tendency to explore the maze. Here, we assessed individual boldness levels in a completely different context by challenging individuals with a novel object to show that this reaction correlates with the tendency to explore a route in the presence of an informed individual. This result is opposite to the findings of Nomakuchi *et al*. [43]. An important difference between our findings and those of Nomakuchi *et al*. [43] is that in their study the demonstrators were always the first to enter the maze, whereas in our study naive individuals did not always wait for the informed demonstrator.

Dominance was not related to individual contribution to group movement decisions in our experiment. Several studies have shown that more dominant individuals have a stronger say in determining group movements (e.g. [6–8]; but see [9]). These examples are primarily from species with strong social group structure, such as several species of monkeys in which the highest-ranked individuals have a strong influence over the behaviour of other individuals. For instance, alpha males in chacma baboons (*Papio ursinus*) were able to steer a group towards a food source where few individuals besides the alpha male were able to consume food [7]. In species with a less strong social group structure, like the barnacle goose, the role of dominance on travel directions is probably less strong, as is also confirmed by an absence of a relation between dominance and leadership in domestic goats [44], cattle and sheep [14].

Next to dominance, motivation also has been shown to be important in collective movements; in particular, individuals with a higher need for resources are predicted to lead groups [12,45,46]. Empirical support comes from studies in fish, where food-deprived fish were seen more often in frontal positions than well-fed fish [10], and plains zebra (*Equus burchellii*), where lactating females were more likely to initiate group movements compared with non-lactating females [11]. Likewise, in African buffalo it is mostly adult females, mainly with offspring, that initiate group movements [3]. In our study, we did not find an effect of body condition on individual contribution to group movement decisions. Most probably the individual differences in terms of energy requirements were small in our experiment, as no individuals were food-deprived, or in a stage where they would face high energy requirements (e.g. moult), explaining the lack of a possible effect of body condition.

In conclusion, personality affected individual contribution to group movement decisions in pairs of geese, with bolder individuals deciding more often by themselves on travel direction as compared with shyer individuals, suggesting that bold individuals have a greater influence over the outcome of collective decisions. The effect of personality disappeared in groups of four individuals, suggesting that individual differences were less important with increasing group size.
